# 2-[2-(2,6-Dichloro­anilino)phen­yl]-*N*-[(2*S*)-2-methyl-3-oxo-8-phenyl-1-thia-4-aza­spiro­[4.5]dec-4-yl]acetamide

**DOI:** 10.1107/S1600536810009803

**Published:** 2010-03-20

**Authors:** Mehmet Akkurt, Mebble Nassozi, Ayşe Kocabalkanlı, Islam Ullah Khan, Shahzad Sharif

**Affiliations:** a Department of Physics, Faculty of Arts and Sciences, Erciyes University, 38039 Kayseri, Turkey; b Department of Pharmaceutical Chemistry, Faculty of Pharmacy, Istanbul University, 34116 Beyazit, Istanbul, Turkey; c Materials Chemistry Laboratory, Department of Chemistry, Government College University, Lahore 54000, Pakistan

## Abstract

In the title compound, C_29_H_29_Cl_2_N_3_O_2_S, the phenyl ring is disordered over two orientations with occupancies of 0.55 (3) and 0.45 (3). The mol­ecular packing in the crystal is stabilized by inter­molecular N—H⋯O inter­actions, linking the mol­ecules into infinite chains along the *c* axis. In addition, there are weak C—H⋯S and C—H⋯π inter­actions.

## Related literature

For general background to chemical modifications of the non-steroidal anti-inflammatory drug diclofenac {[2-(2,6-dichloro­anilino)phen­yl]acetic acid}, see: Amir & Shikha (2004 ▶); Bandarage *et al.* (2000 ▶); Bhandari *et al.* (2008 ▶); Galanakis *et al.* (2004 ▶); Sriram *et al.* (2006 ▶); Wittine *et al.* (2009 ▶).
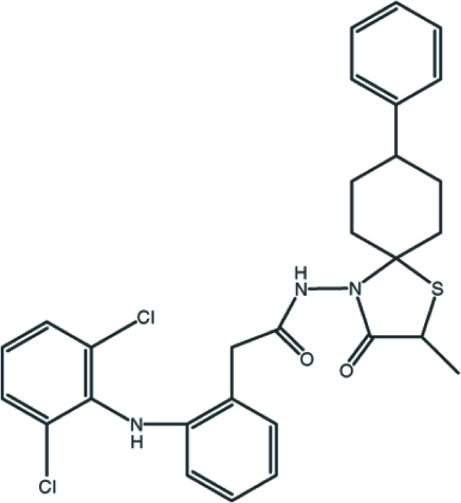


## Experimental

### 

#### Crystal data

C_29_H_29_Cl_2_N_3_O_2_S
*M* *_r_* = 554.52
Monoclinic, 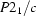

*a* = 11.6105 (6) Å
*b* = 24.3130 (12) Å
*c* = 9.8137 (5) Å
β = 95.335 (2)°
*V* = 2758.3 (2) Å^3^
*Z* = 4
Mo *K*α radiation
μ = 0.34 mm^−1^
*T* = 296 K
0.34 × 0.17 × 0.12 mm

#### Data collection

Bruker APEXII CCD area-detector diffractometer
30871 measured reflections
6792 independent reflections
3599 reflections with *I* > 2σ(*I*)
*R*_int_ = 0.036

#### Refinement

*R*[*F* ^2^ > 2σ(*F* ^2^)] = 0.061
*wR*(*F* ^2^) = 0.216
*S* = 1.03
6792 reflections
380 parameters
29 restraints
H-atom parameters constrained
Δρ_max_ = 0.71 e Å^−3^
Δρ_min_ = −0.39 e Å^−3^

### 

Data collection: *APEX2* (Bruker, 2007 ▶); cell refinement: *SAINT* (Bruker, 2007 ▶); data reduction: *SAINT*; program(s) used to solve structure: *SHELXS97* (Sheldrick, 2008 ▶); program(s) used to refine structure: *SHELXL97* (Sheldrick, 2008 ▶); molecular graphics: *ORTEP-3 for Windows* (Farrugia, 1997 ▶); software used to prepare material for publication: *WinGX* (Farrugia, 1999 ▶) and *PLATON* (Spek, 2009 ▶).

## Supplementary Material

Crystal structure: contains datablocks global, I. DOI: 10.1107/S1600536810009803/bt5217sup1.cif

Structure factors: contains datablocks I. DOI: 10.1107/S1600536810009803/bt5217Isup2.hkl

Additional supplementary materials:  crystallographic information; 3D view; checkCIF report

## Figures and Tables

**Table 1:** Hydrogen-bond geometry (Å, °) *Cg*3 is the centroid of the C7–C12 benzene ring.

*D*—H⋯*A*	*D*—H	H⋯*A*	*D*⋯*A*	*D*—H⋯*A*
N2—H*N*2⋯O1^i^	0.86	2.04	2.795 (3)	146
C20—H20*B*⋯S1	0.97	2.83	3.220 (4)	105
C22—H22*A*⋯S1	0.97	2.84	3.224 (3)	105
C17—H17*A*⋯*Cg*3^ii^	0.96	2.96	3.862 (5)	157

Symmetry codes: (i) 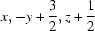; (ii) 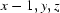.

**Fig. 1.**
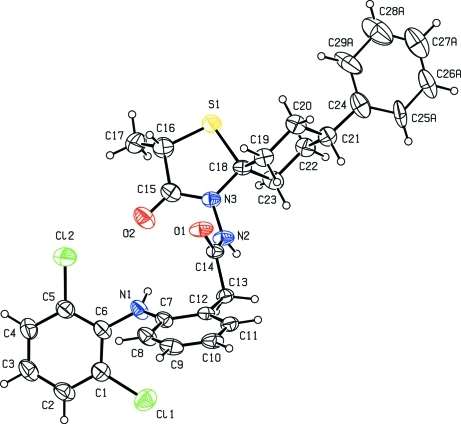
The molecule of the title compound with the atom numbering scheme. Displacement ellipsoids for non-H atoms are drawn at the 30% probability level. The atoms of the minor component of the disorder in the molecule have been omitted.

**Fig. 2.**
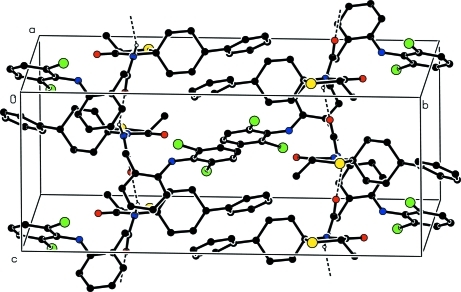
View of the unitcell contents and the hydrogen bonding of the title compound. For the sake of clarity, the atoms of the minor component of the disorder in the molecule and H atoms not involved in hydrogen bonding have been omitted.

## supplementary crystallographic information

### Comment

Diclofenac, [2-(2,6-dichloroanilino)phenyl]acetic acid, is being used for its antiinflammatory activity for over 30 years and is a tolerable drug when compared with other NSAIDs. Still, its chronic use may elicit appreciable GI irritation, bleeding and ulceration due to its free –COOH group. Chemical modifications on the molecule have been made both to improve the safety profile and also to obtain derivatives with antimicrobial, antioxidant and anticancer properties by derivatization of the carboxylate function (Bandarage *et al.*, 2000; Amir & Shikha, 2004; Galanakis *et al.*, 2004; Sriram *et al.*, 2006; Bhandari *et al.*, 2008; Wittine *et al*., 2009). Following the same strategy, we prepared the title molecule bearing a spirothiazolidinone moiety to investigate its antimicrobial potential.

The phenyl ring of the title molecule (Fig. 1) is disordered over two orientations with occupancies of 0.55 (3) and 0.45 (3). The dihedral angle between the planes of the disorder phenyl rings (C24/C25A–C29A and C24/C25B–C29B) is 15.8 (9)°. The two benzene rings (C1–C6 and C7–C12) form dihedral angles of 25.2 (5), 26.6 (8)° and 67.7 (5), 83.4 (8)°, respectively, with these disorder phenyl rings.

Intermolecular N—H···O interactions link the molecules into infinite chains stretching along the *c* axis of the crystal (Fig. 2 and Table 1). In the crystal structure, weak C—H···π interactions occur between the (C17A)H17A atom of the methyl group and the C7–C12 benzene ring (Table 1).

### Experimental

A mixture of 2-[2-(2,6-dichloroanilino)phenyl]-*N*'-(4-phenylcyclohexylidene)acetohydrazide**(0.0025 mol) and 2-mercaptopropionic acid (2.5 ml) was refluxed in dry benzene (20 ml) using a Dean-Stark water separator for 6 h. The reaction mixture thus obtained was concentrated under vacuum and neutralized by addition of saturated NaHCO_3_ solution until CO_2 _evolution ceased. After refrigeration overnight, the precipitate was filtered, dried and purified by recrystallization from EtOH. Yield, 56.1 %, m.p. 492.7-494.3 K. UV (EtOH) λ_max._= 279.6, 205.4 nm. IR (KBr) ν = 3219 (N—H), 1721, 1682 (C=O) cm^-1^. ^1^H-NMR (DMSO-d_6_, 500 MHz) δ= 1.42 (3*H*, d, J= 6.83 Hz, CH_3_), 1.50-1.72 (8*H*, m, CH_2_-sp.*), 2.33 (1*H*, t, J= 12.20 Hz, CH-sp.), 3.74 (2*H*, s, CH_2_CO), 3.93 (1*H*, q, J= 6.83 Hz, SCH), 6.29 (1*H*, d, J= 7.81 Hz, Ar—H*), 6.90 (1*H*, t, J= 6.83 Hz, Ar—H), 7.07 (1*H*, t, J= 7.80 Hz, Ar—H), 7.14-7.20 (4*H*, m, Ar—H and C_6_H_5_-sp.), 7.27 (2*H*, t, J= 7.32 Hz, C_6_H_5_-sp), 7.31 (1*H*, d, J= 7.80 Hz, Ar—H), 7.51 (2*H*, d, J= 7.81 Hz, Ar—H), 7.41 (1*H*, s, NH), 10.51 (1*H*, s, CONH).(sp= spirodecane, Ar= aromatic). Analysis calculated for C_29_H_29_Cl_2_N_3_O_2_S: C 62.81, H 5.27, N 7.58 %. Found: C 62.79, H 5.34, N 7.50 %.

### Refinement

All hydrogen atoms except those of the disordered phenyl ring were located in a difference map. They were refined using a riding model with N—H = 0.86 Å and C—H = 0.93 - 0.98 Å, and  with U_iso_(H) = 1.2U_eq_(C,N) or 1.5U_eq_(C_methyl_). The phenyl ring of the molecule shows a positional disorder over two sites with refined occupancies of 0.55 (3) and 0.45 (3).

### Crystal data

**Table:**

C_29_H_29_Cl_2_N_3_O_2_S	*F*(000) = 1160
*M**_r_* = 554.52	*D*_x_ = 1.335 Mg m^−^^3^
Monoclinic, *P*2_1_/*c*	Mo *K*α radiation, λ = 0.71073 Å
Hall symbol: -P 2ybc	Cell parameters from 6879 reflections
*a* = 11.6105 (6) Å	θ = 2.3–23.7°
*b* = 24.3130 (12) Å	µ = 0.34 mm^−^^1^
*c* = 9.8137 (5) Å	*T* = 296 K
β = 95.335 (2)°	Irregular, off white
*V* = 2758.3 (2) Å^3^	0.34 × 0.17 × 0.12 mm
*Z* = 4	

### Data collection

**Table:**

Bruker APEXII CCD area-detector diffractometer	3599 reflections with *I* > 2σ(*I*)
Radiation source: sealed tube	*R*_int_ = 0.036
graphite	θ_max_ = 28.4°, θ_min_ = 1.8°
φ and ω scans	*h* = −15→15
30871 measured reflections	*k* = −32→32
6792 independent reflections	*l* = −13→13

### Refinement

**Table:**

Refinement on *F*^2^	Primary atom site location: structure-invariant direct methods
Least-squares matrix: full	Secondary atom site location: difference Fourier map
*R*[*F*^2^ > 2σ(*F*^2^)] = 0.061	Hydrogen site location: inferred from neighbouring sites
*wR*(*F*^2^) = 0.216	H-atom parameters constrained
*S* = 1.03	*w* = 1/[σ^2^(*F*_o_^2^) + (0.098*P*)^2^ + 1.278*P*] where *P* = (*F*_o_^2^ + 2*F*_c_^2^)/3
6792 reflections	(Δ/σ)_max_ < 0.001
380 parameters	Δρ_max_ = 0.71 e Å^−^^3^
29 restraints	Δρ_min_ = −0.39 e Å^−^^3^

### Special details

**Table:**

Geometry. Bond distances, angles etc. have been calculated using the rounded fractional coordinates. All su's are estimated from the variances of the (full) variance-covariance matrix. The cell esds are taken into account in the estimation of distances, angles and torsion angles
Refinement. Refinement on *F*^2^ for ALL reflections except those flagged by the user for potential systematic errors. Weighted *R*-factors wR and all goodnesses of fit *S* are based on *F*^2^, conventional *R*-factors *R* are based on *F*, with *F* set to zero for negative *F*^2^. The observed criterion of *F*^2^ > σ(*F*^2^) is used only for calculating -*R*-factor-obs etc. and is not relevant to the choice of reflections for refinement. *R*-factors based on *F*^2^ are statistically about twice as large as those based on *F*, and *R*-factors based on ALL data will be even larger.

### Fractional atomic coordinates and isotropic or equivalent isotropic displacement parameters (Å^2^)

**Table:**

	*x*	*y*	*z*	*U*_iso_*/*U*_eq_	Occ. (<1)
Cl1	0.98524 (9)	0.89260 (5)	0.18049 (12)	0.0906 (4)	
Cl2	0.59184 (11)	0.94402 (5)	−0.12658 (18)	0.1208 (6)	
S1	0.25302 (7)	0.71472 (4)	0.01238 (14)	0.0855 (4)	
O1	0.62380 (18)	0.75690 (11)	−0.1282 (2)	0.0635 (8)	
O2	0.4694 (2)	0.83356 (11)	0.0547 (3)	0.0853 (10)	
N1	0.7725 (2)	0.86657 (10)	−0.0040 (3)	0.0645 (10)	
N2	0.5873 (2)	0.73711 (11)	0.0866 (2)	0.0555 (9)	
N3	0.4692 (2)	0.74017 (11)	0.0565 (3)	0.0523 (8)	
C1	0.8883 (3)	0.94012 (14)	0.1066 (4)	0.0623 (11)	
C2	0.9084 (3)	0.99493 (16)	0.1345 (4)	0.0749 (14)	
C3	0.8308 (4)	1.03345 (16)	0.0839 (4)	0.0788 (16)	
C4	0.7329 (3)	1.01787 (15)	0.0046 (4)	0.0774 (15)	
C5	0.7154 (3)	0.96296 (14)	−0.0258 (4)	0.0671 (11)	
C6	0.7921 (3)	0.92223 (13)	0.0223 (4)	0.0571 (10)	
C7	0.8511 (2)	0.83528 (12)	−0.0746 (3)	0.0499 (10)	
C8	0.9222 (3)	0.85998 (14)	−0.1622 (4)	0.0649 (11)	
C9	1.0001 (3)	0.82913 (17)	−0.2277 (4)	0.0680 (13)	
C10	1.0088 (3)	0.77434 (16)	−0.2055 (4)	0.0641 (13)	
C11	0.9380 (2)	0.74935 (13)	−0.1189 (3)	0.0556 (10)	
C12	0.8588 (2)	0.77898 (12)	−0.0517 (3)	0.0441 (9)	
C13	0.7841 (2)	0.75166 (13)	0.0447 (3)	0.0510 (9)	
C14	0.6583 (2)	0.74927 (12)	−0.0099 (3)	0.0450 (9)	
C15	0.4177 (3)	0.79046 (15)	0.0432 (4)	0.0635 (11)	
C16	0.2869 (3)	0.78542 (16)	0.0060 (5)	0.0794 (16)	
C17	0.2194 (4)	0.82223 (18)	0.0830 (6)	0.103 (2)	
C18	0.4014 (2)	0.68929 (12)	0.0445 (3)	0.0477 (9)	
C19	0.4340 (3)	0.65415 (13)	−0.0732 (3)	0.0551 (10)	
C20	0.3638 (3)	0.60153 (14)	−0.0850 (3)	0.0627 (11)	
C21	0.3770 (3)	0.56817 (14)	0.0470 (4)	0.0636 (11)	
C22	0.3460 (3)	0.60400 (14)	0.1664 (3)	0.0624 (11)	
C23	0.4159 (3)	0.65690 (14)	0.1768 (3)	0.0615 (11)	
C24	0.3100 (4)	0.51492 (16)	0.0429 (5)	0.0862 (18)	
C25A	0.3440 (11)	0.4680 (6)	0.1147 (16)	0.085 (4)	0.55 (3)
C26A	0.2744 (18)	0.4224 (3)	0.113 (2)	0.101 (7)	0.55 (3)
C27A	0.167 (2)	0.4217 (5)	0.0386 (15)	0.114 (7)	0.55 (3)
C28A	0.1244 (19)	0.4675 (9)	−0.0298 (17)	0.151 (8)	0.55 (3)
C29A	0.1961 (13)	0.5155 (6)	−0.0263 (17)	0.127 (6)	0.55 (3)
C27B	0.230 (3)	0.4101 (6)	0.064 (3)	0.152 (12)	0.45 (3)
C28B	0.178 (3)	0.4464 (13)	−0.030 (3)	0.22 (2)	0.45 (3)
C29B	0.221 (2)	0.4988 (9)	−0.044 (2)	0.175 (12)	0.45 (3)
C25B	0.3636 (13)	0.4808 (7)	0.1432 (17)	0.115 (7)	0.45 (3)
C26B	0.322 (2)	0.4272 (5)	0.155 (2)	0.139 (10)	0.45 (3)
H3	0.84430	1.07050	0.10320	0.0940*	
H9	1.04690	0.84610	−0.28730	0.0810*	
H4	0.67890	1.04400	−0.02840	0.0930*	
H8	0.91740	0.89770	−0.17710	0.0780*	
H13A	0.79090	0.77150	0.13080	0.0610*	
H13B	0.81210	0.71450	0.06270	0.0610*	
H16A	0.27270	0.79640	−0.09010	0.0950*	
H17A	0.13870	0.81750	0.05430	0.1540*	
H17B	0.24140	0.85960	0.06700	0.1540*	
H10	1.06230	0.75360	−0.24840	0.0770*	
H11	0.94360	0.71150	−0.10540	0.0670*	
H19B	0.42110	0.67480	−0.15780	0.0660*	
H20A	0.38880	0.57940	−0.15910	0.0750*	
H20B	0.28290	0.61050	−0.10710	0.0750*	
H21	0.45910	0.55870	0.06460	0.0760*	
H22A	0.26430	0.61290	0.15420	0.0750*	
H22B	0.36020	0.58350	0.25110	0.0750*	
H23A	0.39120	0.67930	0.25060	0.0740*	
H23B	0.49710	0.64810	0.19850	0.0740*	
H25A	0.41590	0.46730	0.16520	0.1020*	0.55 (3)
H26A	0.29950	0.39150	0.16350	0.1210*	0.55 (3)
H27A	0.12350	0.38960	0.03480	0.1370*	0.55 (3)
H28A	0.05110	0.46750	−0.07690	0.1810*	0.55 (3)
H29A	0.16810	0.54750	−0.06960	0.1530*	0.55 (3)
H17C	0.23330	0.81410	0.17890	0.1540*	
H19A	0.51570	0.64510	−0.05930	0.0660*	
H1	0.71200	0.85100	0.02280	0.0780*	
HN2	0.61570	0.72740	0.16710	0.0670*	
H2	0.97490	1.00560	0.18800	0.0900*	
H25B	0.42610	0.49330	0.20120	0.1380*	0.45 (3)
H26B	0.35410	0.40360	0.22310	0.1650*	0.45 (3)
H27B	0.20380	0.37390	0.06550	0.1830*	0.45 (3)
H28B	0.11210	0.43550	−0.08490	0.2640*	0.45 (3)
H29B	0.18840	0.52230	−0.11210	0.2100*	0.45 (3)

### Atomic displacement parameters (Å^2^)

**Table:**

	*U*^11^	*U*^22^	*U*^33^	*U*^12^	*U*^13^	*U*^23^
Cl1	0.0807 (7)	0.0962 (8)	0.0934 (8)	0.0224 (5)	0.0001 (6)	−0.0116 (6)
Cl2	0.0885 (8)	0.0901 (8)	0.1737 (14)	0.0009 (6)	−0.0423 (8)	−0.0296 (8)
S1	0.0438 (4)	0.0637 (6)	0.1487 (11)	0.0024 (4)	0.0068 (5)	−0.0140 (6)
O1	0.0474 (11)	0.1089 (18)	0.0334 (11)	−0.0139 (11)	−0.0010 (9)	0.0081 (11)
O2	0.0841 (17)	0.0699 (16)	0.105 (2)	−0.0231 (14)	0.0252 (15)	−0.0047 (15)
N1	0.0560 (15)	0.0461 (14)	0.096 (2)	−0.0102 (11)	0.0310 (15)	−0.0135 (14)
N2	0.0434 (13)	0.0856 (18)	0.0367 (13)	−0.0132 (12)	−0.0007 (10)	0.0080 (12)
N3	0.0427 (12)	0.0654 (16)	0.0492 (15)	−0.0081 (11)	0.0069 (11)	0.0030 (12)
C1	0.0593 (19)	0.063 (2)	0.067 (2)	0.0010 (15)	0.0184 (17)	−0.0100 (16)
C2	0.069 (2)	0.070 (2)	0.086 (3)	−0.0117 (19)	0.009 (2)	−0.025 (2)
C3	0.088 (3)	0.056 (2)	0.095 (3)	−0.0124 (19)	0.022 (2)	−0.023 (2)
C4	0.082 (3)	0.0510 (19)	0.099 (3)	0.0018 (17)	0.008 (2)	−0.0074 (19)
C5	0.0622 (19)	0.0591 (19)	0.080 (2)	−0.0057 (16)	0.0061 (17)	−0.0102 (17)
C6	0.0548 (17)	0.0496 (17)	0.070 (2)	−0.0072 (14)	0.0231 (16)	−0.0128 (16)
C7	0.0422 (14)	0.0510 (16)	0.0583 (19)	−0.0077 (12)	0.0145 (13)	−0.0086 (14)
C8	0.0619 (19)	0.0583 (19)	0.077 (2)	−0.0173 (15)	0.0195 (18)	−0.0074 (17)
C9	0.0492 (17)	0.095 (3)	0.062 (2)	−0.0205 (17)	0.0162 (16)	−0.0116 (19)
C10	0.0367 (15)	0.087 (3)	0.069 (2)	0.0002 (15)	0.0064 (15)	−0.0250 (19)
C11	0.0400 (15)	0.0591 (18)	0.066 (2)	0.0044 (13)	−0.0041 (14)	−0.0133 (15)
C12	0.0335 (12)	0.0517 (16)	0.0460 (16)	−0.0054 (11)	−0.0013 (11)	−0.0062 (12)
C13	0.0429 (15)	0.0582 (17)	0.0501 (17)	−0.0044 (13)	−0.0059 (13)	0.0065 (14)
C14	0.0424 (14)	0.0562 (16)	0.0359 (15)	−0.0081 (12)	0.0006 (12)	0.0031 (13)
C15	0.0611 (19)	0.070 (2)	0.062 (2)	−0.0110 (17)	0.0199 (16)	−0.0037 (17)
C16	0.060 (2)	0.080 (3)	0.101 (3)	−0.0003 (18)	0.022 (2)	0.004 (2)
C17	0.074 (3)	0.080 (3)	0.151 (5)	0.020 (2)	−0.012 (3)	−0.021 (3)
C18	0.0371 (13)	0.0624 (17)	0.0448 (17)	−0.0053 (12)	0.0096 (12)	−0.0045 (14)
C19	0.0565 (17)	0.070 (2)	0.0404 (16)	−0.0002 (15)	0.0124 (14)	−0.0008 (15)
C20	0.068 (2)	0.071 (2)	0.0510 (19)	−0.0034 (16)	0.0161 (16)	−0.0150 (16)
C21	0.0618 (19)	0.062 (2)	0.069 (2)	0.0014 (15)	0.0163 (17)	−0.0030 (17)
C22	0.067 (2)	0.071 (2)	0.0505 (19)	−0.0101 (16)	0.0120 (16)	0.0059 (16)
C23	0.071 (2)	0.075 (2)	0.0405 (17)	−0.0179 (17)	0.0159 (15)	−0.0001 (15)
C24	0.117 (4)	0.061 (2)	0.087 (3)	−0.014 (2)	0.043 (3)	−0.013 (2)
C25A	0.101 (7)	0.026 (5)	0.138 (10)	0.007 (4)	0.063 (6)	0.005 (6)
C26A	0.124 (15)	0.043 (6)	0.148 (14)	−0.014 (6)	0.074 (11)	−0.010 (8)
C27A	0.162 (15)	0.082 (9)	0.110 (10)	−0.028 (9)	0.074 (10)	−0.029 (7)
C28A	0.216 (17)	0.145 (15)	0.090 (9)	−0.094 (14)	0.000 (9)	0.009 (9)
C29A	0.177 (13)	0.110 (10)	0.086 (8)	−0.092 (10)	−0.032 (9)	0.015 (9)
C27B	0.15 (2)	0.068 (9)	0.25 (3)	−0.053 (12)	0.09 (2)	−0.076 (13)
C28B	0.28 (4)	0.14 (2)	0.23 (4)	−0.12 (3)	−0.04 (3)	−0.04 (2)
C29B	0.28 (3)	0.102 (12)	0.138 (17)	−0.092 (15)	−0.001 (17)	−0.053 (12)
C25B	0.137 (11)	0.052 (9)	0.170 (15)	−0.031 (8)	0.091 (11)	−0.050 (10)
C26B	0.126 (17)	0.097 (12)	0.21 (2)	−0.031 (9)	0.106 (14)	−0.071 (13)

### Geometric parameters (Å, °)

**Table:**

Cl1—C1	1.725 (4)	C25A—C26A	1.37 (2)
Cl2—C5	1.728 (4)	C25B—C26B	1.40 (2)
S1—C16	1.766 (4)	C26A—C27A	1.39 (3)
S1—C18	1.830 (3)	C26B—C27B	1.39 (4)
O1—C14	1.207 (4)	C27A—C28A	1.37 (3)
O2—C15	1.208 (4)	C27B—C28B	1.38 (4)
N1—C6	1.392 (4)	C28A—C29A	1.43 (3)
N1—C7	1.418 (4)	C28B—C29B	1.38 (4)
N2—N3	1.378 (3)	C2—H2	0.9300
N2—C14	1.345 (3)	C3—H3	0.9300
N3—C15	1.362 (4)	C4—H4	0.9300
N3—C18	1.465 (4)	C8—H8	0.9300
N1—H1	0.8600	C9—H9	0.9300
N2—HN2	0.8600	C10—H10	0.9300
C1—C2	1.376 (5)	C11—H11	0.9300
C1—C6	1.396 (5)	C13—H13A	0.9700
C2—C3	1.361 (6)	C13—H13B	0.9700
C3—C4	1.370 (6)	C16—H16A	0.9800
C4—C5	1.379 (5)	C17—H17A	0.9600
C5—C6	1.385 (5)	C17—H17B	0.9600
C7—C8	1.383 (5)	C17—H17C	0.9600
C7—C12	1.389 (4)	C19—H19A	0.9700
C8—C9	1.379 (5)	C19—H19B	0.9700
C9—C10	1.352 (6)	C20—H20A	0.9700
C10—C11	1.377 (5)	C20—H20B	0.9700
C11—C12	1.383 (4)	C21—H21	0.9800
C12—C13	1.497 (4)	C22—H22A	0.9700
C13—C14	1.509 (3)	C22—H22B	0.9700
C15—C16	1.534 (5)	C23—H23A	0.9700
C16—C17	1.448 (6)	C23—H23B	0.9700
C18—C19	1.513 (4)	C25A—H25A	0.9300
C18—C23	1.515 (4)	C25B—H25B	0.9300
C19—C20	1.516 (5)	C26A—H26A	0.9300
C20—C21	1.524 (5)	C26B—H26B	0.9300
C21—C24	1.509 (5)	C27A—H27A	0.9300
C21—C22	1.530 (5)	C27B—H27B	0.9300
C22—C23	1.519 (5)	C28A—H28A	0.9300
C24—C29A	1.429 (16)	C28B—H28B	0.9300
C24—C25A	1.379 (15)	C29A—H29A	0.9300
C24—C25B	1.389 (17)	C29B—H29B	0.9300
C24—C29B	1.34 (2)		
			
Cl1···N1	2.991 (3)	C29B···H20B	2.8900
Cl1···C7	3.148 (3)	C29B···H20A	3.0500
Cl1···C8	3.467 (4)	H1···Cl2	2.9700
Cl1···C17^i^	3.423 (5)	H1···O2	2.8900
Cl1···C27A^ii^	3.483 (18)	H1···C13	2.5600
Cl2···N1	2.987 (3)	H1···O1	2.8600
Cl2···C22^iii^	3.544 (4)	H1···H13A	2.3500
Cl2···C23^iii^	3.631 (4)	H1···C14	2.5600
Cl1···H17A^i^	2.9100	HN2···H23B	2.4100
Cl2···H1	2.9700	HN2···H13A	2.3600
Cl2···H22B^iii^	2.9200	HN2···C23	2.8900
Cl2···H23B^iii^	2.9700	HN2···O1^v^	2.0400
Cl2···H25B^iii^	2.8800	H2···H28A^xii^	2.4800
S1···N3	2.582 (3)	H3···H17B^ix^	2.5200
S1···C10^iv^	3.686 (4)	H4···H17B^ix^	2.5600
S1···H20B	2.8300	H8···H28B^x^	2.5000
S1···H22A	2.8400	H8···C1	3.0200
O1···N3	2.698 (3)	H8···C6	2.6100
O1···C7	3.258 (3)	H11···H13B	2.3500
O1···C15	3.158 (4)	H13A···C11^v^	2.9000
O1···C19	3.407 (4)	H13A···N1	2.6600
O1···N2^iii^	2.795 (3)	H13A···H1	2.3500
O2···N2	2.719 (4)	H13A···C10^v^	3.0800
O2···C14	3.110 (4)	H13A···HN2	2.3600
O1···HN2^iii^	2.0400	H13B···C9^v^	3.0500
O1···H1	2.8600	H13B···C10^v^	3.0800
O2···H1	2.8900	H13B···H27B^xi^	2.4900
O2···H17B	2.7400	H13B···H11	2.3500
N1···Cl1	2.991 (3)	H16A···H23A^iii^	2.2600
N1···Cl2	2.987 (3)	H17A···C9^iv^	3.0800
N1···C14	3.143 (4)	H17A···Cl1^iv^	2.9100
N2···O2	2.719 (4)	H17A···C10^iv^	3.0300
N2···O1^v^	2.795 (3)	H17B···C4^ix^	3.0800
N3···O1	2.698 (3)	H17B···O2	2.7400
N3···S1	2.582 (3)	H17B···C3^ix^	3.0700
N1···H13A	2.6600	H17B···H4^ix^	2.5600
N2···H19A	2.7400	H17B···H3^ix^	2.5200
N2···H23B	2.6800	H17C···H19B^v^	2.6000
C1···C8	3.332 (5)	H19A···H23B	2.5600
C1···C26A^ii^	3.51 (2)	H19A···C26A^xi^	3.0200
C1···C26B^ii^	3.55 (2)	H19A···N2	2.7400
C2···C2^vi^	3.549 (5)	H19A···C14	3.0400
C6···C26B^ii^	3.55 (2)	H19A···H21	2.5400
C7···Cl1	3.148 (3)	H19A···C26B^xi^	2.8000
C7···O1	3.258 (3)	H19A···H26B^xi^	2.5900
C8···C1	3.332 (5)	H19B···C15^iii^	3.0500
C8···Cl1	3.467 (4)	H19B···H17C^iii^	2.6000
C10···C13^iii^	3.468 (5)	H20A···H25A^xi^	2.5400
C10···S1^i^	3.686 (4)	H20A···C29B	3.0500
C13···C10^v^	3.468 (5)	H20B···S1	2.8300
C14···C19	3.495 (4)	H20B···H29B	2.4100
C14···N1	3.143 (4)	H20B···H22A	2.5900
C14···O2	3.110 (4)	H20B···H29A	2.0900
C15···O1	3.158 (4)	H20B···C29A	2.6700
C17···Cl1^iv^	3.423 (5)	H20B···C29B	2.8900
C19···O1	3.407 (4)	H21···H25A	2.5000
C19···C14	3.495 (4)	H21···H25B	2.1400
C22···Cl2^v^	3.544 (4)	H21···H23B	2.5600
C23···Cl2^v^	3.631 (4)	H21···H19A	2.5400
C26A···C1^vii^	3.51 (2)	H21···C25A^xi^	3.0800
C26B···C6^vii^	3.55 (2)	H22A···C29A	3.0200
C26B···C1^vii^	3.55 (2)	H22A···S1	2.8400
C27A···Cl1^vii^	3.483 (18)	H22A···H20B	2.5900
C28A···C28A^viii^	3.39 (3)	H22B···Cl2^v^	2.9200
C1···H8	3.0200	H22B···C25B	2.7100
C3···H17B^ix^	3.0700	H22B···H25B	2.3900
C4···H17B^ix^	3.0800	H23A···C16^v^	3.0100
C6···H8	2.6100	H23A···H16A^v^	2.2600
C8···H28B^x^	3.0800	H23A···C15^v^	2.9500
C9···H13B^iii^	3.0500	H23B···H21	2.5600
C9···H17A^i^	3.0800	H23B···Cl2^v^	2.9700
C10···H17A^i^	3.0300	H23B···N2	2.6800
C10···H13A^iii^	3.0800	H23B···HN2	2.4100
C10···H13B^iii^	3.0800	H23B···H19A	2.5600
C11···H13A^iii^	2.9000	H25A···H21	2.5000
C13···H1	2.5600	H25A···H20A^xi^	2.5400
C14···H1	2.5600	H25B···C22	2.8600
C14···H19A	3.0400	H25B···Cl2^v^	2.8800
C15···H23A^iii^	2.9500	H25B···H21	2.1400
C15···H19B^v^	3.0500	H25B···H22B	2.3900
C16···H23A^iii^	3.0100	H26B···H19A^xi^	2.5900
C20···H29B	2.8000	H27B···H13B^xi^	2.4900
C20···H29A	2.6400	H28A···H2^xiii^	2.4800
C22···H25B	2.8600	H28A···H28A^viii^	2.5500
C23···HN2	2.8900	H28A···C28A^viii^	2.8500
C25A···H21^xi^	3.0800	H28B···C8^xiv^	3.0800
C25B···H22B	2.7100	H28B···H8^xiv^	2.5000
C26A···H19A^xi^	3.0200	H29A···H20B	2.0900
C26B···H19A^xi^	2.8000	H29A···C20	2.6400
C28A···H28A^viii^	2.8500	H29B···C20	2.8000
C29A···H22A	3.0200	H29B···H20B	2.4100
C29A···H20B	2.6700		
			
C16—S1—C18	97.22 (15)	C1—C2—H2	120.00
C6—N1—C7	120.7 (3)	C3—C2—H2	120.00
N3—N2—C14	120.0 (2)	C2—C3—H3	120.00
N2—N3—C15	119.2 (3)	C4—C3—H3	120.00
N2—N3—C18	119.2 (2)	C3—C4—H4	120.00
C15—N3—C18	121.5 (2)	C5—C4—H4	120.00
C7—N1—H1	120.00	C7—C8—H8	120.00
C6—N1—H1	120.00	C9—C8—H8	120.00
N3—N2—HN2	120.00	C8—C9—H9	120.00
C14—N2—HN2	120.00	C10—C9—H9	120.00
Cl1—C1—C6	119.7 (3)	C9—C10—H10	120.00
Cl1—C1—C2	118.2 (3)	C11—C10—H10	120.00
C2—C1—C6	122.1 (3)	C10—C11—H11	119.00
C1—C2—C3	120.0 (4)	C12—C11—H11	119.00
C2—C3—C4	120.2 (4)	C12—C13—H13A	109.00
C3—C4—C5	119.2 (3)	C12—C13—H13B	109.00
Cl2—C5—C4	118.7 (3)	C14—C13—H13A	109.00
Cl2—C5—C6	118.6 (3)	C14—C13—H13B	109.00
C4—C5—C6	122.8 (3)	H13A—C13—H13B	108.00
N1—C6—C1	121.4 (3)	S1—C16—H16A	106.00
N1—C6—C5	122.9 (3)	C15—C16—H16A	106.00
C1—C6—C5	115.7 (3)	C17—C16—H16A	106.00
N1—C7—C12	119.0 (2)	C16—C17—H17A	109.00
C8—C7—C12	119.7 (3)	C16—C17—H17B	110.00
N1—C7—C8	121.3 (3)	C16—C17—H17C	110.00
C7—C8—C9	120.6 (3)	H17A—C17—H17B	109.00
C8—C9—C10	120.2 (3)	H17A—C17—H17C	109.00
C9—C10—C11	119.6 (3)	H17B—C17—H17C	109.00
C10—C11—C12	121.7 (3)	C18—C19—H19A	109.00
C7—C12—C13	120.5 (2)	C18—C19—H19B	109.00
C11—C12—C13	121.4 (3)	C20—C19—H19A	109.00
C7—C12—C11	118.2 (3)	C20—C19—H19B	109.00
C12—C13—C14	113.0 (2)	H19A—C19—H19B	108.00
O1—C14—N2	122.7 (2)	C19—C20—H20A	109.00
O1—C14—C13	124.0 (2)	C19—C20—H20B	109.00
N2—C14—C13	113.3 (2)	C21—C20—H20A	109.00
O2—C15—C16	124.4 (3)	C21—C20—H20B	109.00
N3—C15—C16	111.6 (3)	H20A—C20—H20B	108.00
O2—C15—N3	124.1 (3)	C20—C21—H21	107.00
S1—C16—C15	106.8 (3)	C22—C21—H21	107.00
C15—C16—C17	113.5 (4)	C24—C21—H21	107.00
S1—C16—C17	116.8 (3)	C21—C22—H22A	109.00
N3—C18—C19	111.6 (2)	C21—C22—H22B	109.00
S1—C18—C23	110.6 (2)	C23—C22—H22A	109.00
S1—C18—N3	102.64 (19)	C23—C22—H22B	109.00
N3—C18—C23	110.7 (2)	H22A—C22—H22B	108.00
C19—C18—C23	110.2 (2)	C18—C23—H23A	109.00
S1—C18—C19	111.0 (2)	C18—C23—H23B	109.00
C18—C19—C20	111.4 (3)	C22—C23—H23A	109.00
C19—C20—C21	111.9 (3)	C22—C23—H23B	109.00
C20—C21—C22	109.6 (3)	H23A—C23—H23B	108.00
C20—C21—C24	115.0 (3)	C24—C25A—H25A	119.00
C22—C21—C24	110.6 (3)	C26A—C25A—H25A	119.00
C21—C22—C23	111.8 (3)	C24—C25B—H25B	121.00
C18—C23—C22	111.5 (2)	C26B—C25B—H25B	121.00
C21—C24—C25A	125.0 (7)	C25A—C26A—H26A	119.00
C25B—C24—C29B	123.1 (12)	C27A—C26A—H26A	119.00
C21—C24—C29A	117.1 (7)	C25B—C26B—H26B	121.00
C21—C24—C25B	107.3 (7)	C27B—C26B—H26B	121.00
C21—C24—C29B	129.4 (10)	C26A—C27A—H27A	119.00
C25A—C24—C29A	117.3 (9)	C28A—C27A—H27A	120.00
C24—C25A—C26A	121.5 (13)	C26B—C27B—H27B	120.00
C24—C25B—C26B	118.7 (14)	C28B—C27B—H27B	120.00
C25A—C26A—C27A	121.1 (13)	C27A—C28A—H28A	121.00
C25B—C26B—C27B	118.4 (16)	C29A—C28A—H28A	121.00
C26A—C27A—C28A	121.0 (15)	C27B—C28B—H28B	119.00
C26B—C27B—C28B	120.1 (19)	C29B—C28B—H28B	119.00
C27A—C28A—C29A	117.9 (18)	C24—C29A—H29A	120.00
C27B—C28B—C29B	121 (3)	C28A—C29A—H29A	120.00
C24—C29A—C28A	120.9 (13)	C24—C29B—H29B	121.00
C24—C29B—C28B	118 (2)	C28B—C29B—H29B	121.00
			
C16—S1—C18—N3	3.3 (3)	C12—C7—C8—C9	0.4 (5)
C16—S1—C18—C19	−116.0 (3)	C8—C7—C12—C11	−0.3 (4)
C16—S1—C18—C23	121.5 (3)	C8—C7—C12—C13	178.9 (3)
C18—S1—C16—C17	−133.4 (4)	C7—C8—C9—C10	−0.9 (6)
C18—S1—C16—C15	−5.3 (3)	C8—C9—C10—C11	1.1 (6)
C7—N1—C6—C1	−63.7 (5)	C9—C10—C11—C12	−1.0 (5)
C6—N1—C7—C12	153.6 (3)	C10—C11—C12—C13	−178.6 (3)
C7—N1—C6—C5	120.2 (4)	C10—C11—C12—C7	0.6 (4)
C6—N1—C7—C8	−24.5 (5)	C7—C12—C13—C14	70.6 (3)
C14—N2—N3—C15	−73.3 (4)	C11—C12—C13—C14	−110.2 (3)
C14—N2—N3—C18	108.9 (3)	C12—C13—C14—O1	15.6 (4)
N3—N2—C14—O1	−8.8 (5)	C12—C13—C14—N2	−165.1 (3)
N3—N2—C14—C13	171.9 (3)	N3—C15—C16—S1	5.9 (4)
C18—N3—C15—O2	178.6 (3)	N3—C15—C16—C17	135.9 (4)
C18—N3—C15—C16	−3.8 (5)	O2—C15—C16—S1	−176.5 (3)
N2—N3—C18—S1	177.5 (2)	O2—C15—C16—C17	−46.5 (6)
C15—N3—C18—C19	118.7 (3)	S1—C18—C19—C20	−66.3 (3)
C15—N3—C18—C23	−118.2 (3)	N3—C18—C19—C20	179.9 (2)
N2—N3—C18—C23	59.5 (3)	C23—C18—C19—C20	56.5 (3)
C15—N3—C18—S1	−0.2 (4)	S1—C18—C23—C22	66.8 (3)
N2—N3—C18—C19	−63.6 (3)	N3—C18—C23—C22	179.9 (3)
N2—N3—C15—C16	178.5 (3)	C19—C18—C23—C22	−56.2 (3)
N2—N3—C15—O2	0.9 (5)	C18—C19—C20—C21	−56.9 (4)
C6—C1—C2—C3	2.7 (6)	C19—C20—C21—C22	55.0 (4)
Cl1—C1—C2—C3	−176.6 (3)	C19—C20—C21—C24	−179.7 (3)
C2—C1—C6—C5	−3.1 (6)	C20—C21—C22—C23	−54.6 (4)
Cl1—C1—C6—N1	−0.1 (5)	C24—C21—C22—C23	177.6 (3)
Cl1—C1—C6—C5	176.3 (3)	C20—C21—C24—C25A	148.6 (8)
C2—C1—C6—N1	−179.5 (3)	C20—C21—C24—C29A	−40.0 (9)
C1—C2—C3—C4	−0.3 (6)	C22—C21—C24—C25A	−86.6 (9)
C2—C3—C4—C5	−1.5 (6)	C22—C21—C24—C29A	84.8 (8)
C3—C4—C5—C6	1.0 (6)	C21—C22—C23—C18	56.1 (4)
C3—C4—C5—Cl2	−179.7 (3)	C21—C24—C25A—C26A	175.4 (12)
Cl2—C5—C6—N1	−1.8 (5)	C29A—C24—C25A—C26A	4.0 (19)
C4—C5—C6—N1	177.5 (3)	C21—C24—C29A—C28A	−177.1 (12)
C4—C5—C6—C1	1.2 (6)	C25A—C24—C29A—C28A	−5.0 (19)
Cl2—C5—C6—C1	−178.1 (3)	C24—C25A—C26A—C27A	0(3)
N1—C7—C12—C11	−178.4 (3)	C25A—C26A—C27A—C28A	−4(3)
N1—C7—C12—C13	0.8 (4)	C26A—C27A—C28A—C29A	2(3)
N1—C7—C8—C9	178.5 (3)	C27A—C28A—C29A—C24	2(2)

Symmetry codes: (i) *x*+1, *y*, *z*; (ii) −*x*+1, *y*+1/2, −*z*+1/2; (iii) *x*, −*y*+3/2, *z*−1/2; (iv) *x*−1, *y*, *z*; (v) *x*, −*y*+3/2, *z*+1/2; (vi) −*x*+2, −*y*+2, −*z*; (vii) −*x*+1, *y*−1/2, −*z*+1/2; (viii) −*x*, −*y*+1, −*z*; (ix) −*x*+1, −*y*+2, −*z*; (x) −*x*+1, *y*+1/2, −*z*−1/2; (xi) −*x*+1, −*y*+1, −*z*; (xii) *x*+1, −*y*+3/2, *z*+1/2; (xiii) *x*−1, −*y*+3/2, *z*−1/2; (xiv) −*x*+1, *y*−1/2, −*z*−1/2.

### Hydrogen-bond geometry (Å, °)

**Table:**

Cg3 is the centroid of the C7–C12 benzene ring.

**Table:**

*D*—H···*A*	*D*—H	H···*A*	*D*···*A*	*D*—H···*A*
N2—HN2···O1^v^	0.86	2.04	2.795 (3)	146
C20—H20B···S1	0.97	2.83	3.220 (4)	105
C22—H22A···S1	0.97	2.84	3.224 (3)	105
C17—H17A···Cg3^iv^	0.96	2.96	3.862 (5)	157

Symmetry codes: (v) *x*, −*y*+3/2, *z*+1/2; (iv) *x*−1, *y*, *z*.
